# Heart Rate and Perceived Experience Differ Markedly for Children in Same- versus Mixed-Gender Soccer Played as Small- and Large-Sided Games

**DOI:** 10.1155/2018/7804642

**Published:** 2018-08-05

**Authors:** S. Póvoas, M. B. Randers, P. Krustrup, M. N. Larsen, R. Pereira, C. Castagna

**Affiliations:** ^1^Research Center in Sports Sciences, Health Sciences and Human Development, CIDESD, University Institute of Maia, ISMAI, Maia, Portugal; ^2^Department of Sports Science and Clinical Biomechanics, SDU Sport and Health Sciences Cluster (SHSC), University of Southern Denmark, Odense, Denmark; ^3^Research Center in Physical Activity, Health and Leisure, Faculty of Sport, University of Porto, Porto, Portugal; ^4^Fitness Training and Biomechanics Laboratory, Italian Football Federation (FIGC), Technical Department, Coverciano, Florence, Italy; ^5^School of Sport and Exercise Sciences, University of Rome Tor Vergata, Rome, Italy

## Abstract

This study examines heart rate (HR) and perceived experience during same- versus mixed-gender soccer played as small- (SSG) and large-sided (LSG) games. HR, rating of perceived exertion (RPE), and fun scores were determined in 134 pupils (50 girls, 84 boys) randomly assigned to same- and mixed-genders formats playing 2x15-min of SSG (2v2, 4v4) and LSG (12v12) in a random order (~50 m^2^/player). HR was lower (*p*≤0.03) for girls when playing together with boys than when playing alone (71±10 versus 77±7%HRmax), while being similar for boys playing mixed- or same-gender games (74±7 versus 77±4%HRmax). Boys perceived less fun when playing together with girls than when playing alone (4.4±2.3 versus 6.3±2.3,* p*<0.001). Irrespective of gender, higher (*p*<0.001) HRmean, %time>80%HRmax, and RPE were observed during 2v2 (78±9%HRmax, 43±33%, 5.5±2.5) and 4v4 (76±9%HRmax, 39±32%, 5.5±2.7) than during 12v12 (70±10%HRmax, 23±27%, 3.8±2.9). Cardiovascular strain was lower for girls when playing together with boys than when playing alone in LSG. SSG were more intense than LSG when girls played mixed-gender games and when boys played mixed- and same-gender games. When boys played mixed-gender games, SSG were considered more fun than LSG. Physical education teachers and coaches should consider gender and game format differences when using soccer.

## 1. Introduction

It is well established that physical activity is a cornerstone in the prevention of lifestyle diseases [1], and there is increasing evidence that sports participation has the potential to improve the health of nations [2]. In recent years, soccer played as small-sided games has been established as a health-promoting and performance-enhancing activity in several populations [3, 4]. Small-sided games have been shown to have a major impact on bones and muscles due to numerous demanding actions, as well as on the cardiovascular system by imposing high heart rates (HR), thus acting as broad-spectrum prevention of lifestyle diseases [5].

Population-based studies have indicated that the guidelines of 60 min of moderate-to-vigorous physical activity every day are not met by many children and adolescents [6, 7]. Moreover, a gender-based disparity in physical activity is observed among young people, with girls performing less moderate-to-vigorous physical activity than boys [8–11]. Considerable attention has therefore been paid to identifying gender-sensitive settings in which children and adolescents can engage in moderate-to-vigorous physical activity on a regular basis in order to enhance their health profile [12].

School offers an opportunity to interact with most children and adolescents, and soccer is a part of the physical education (PE) curriculum of most grades in many countries around the globe. Several recent studies have shown that small-sided soccer games in school settings can improve the health profile of children and adolescents [13–16]. In these studies, both genders played together and marked improvements were found in both boys and girls. Nevertheless, only one study [16] reported results in relation to gender and no significant differences in HR response were found between boys and girls or between pupils who were active in sports clubs and those who were not.

Many factors have been shown to influence the activity profile and physiological response to small-sided games (e.g., number of players) [17], but most studies focus on male adults or elite boys. Information on the demands of different game formats in school settings for untrained children and adolescents of both genders is limited to 3v3 games [16, 18, 19]. It is not therefore known what is the best soccer game format for promoting high HRs in each gender.

In school, boys and girls play together, but information on the demands of different game formats in school settings for children and adolescents of both genders is scarce and, as far as we know, no study has investigated the effects of gender format (mixed- or same-gender) on intensity and perceived experience during soccer training.

Since girls engage less in moderate-to-vigorous activity than boys [8–11], attention should be paid to increasing the level of participation of this group in this type of activity. Traditionally, more boys play soccer in their leisure time than girls [20], and generally boys are more experienced in soccer. It might therefore be imagined that there is a risk of boys dominating during mixed-gender games.

Thus, the purpose of this study is to describe HR response and perceived experience in different soccer gender (same versus mixed) and game formats (2v2, 4v4, and 12v12, i.e., small- versus large-sided games) for adolescent girls and boys during PE lessons. It was hypothesized that there would be practical gender and format differences in the cardiovascular load and perceived experience of the activity.

## 2. Materials and Methods

### 2.1. Subjects

A total of 134 pupils (50 girls, 84 boys) aged 12-16 years from a secondary school in the Porto District (Portugal) were randomly chosen to participate in this study. The descriptive characteristics of the participants by gender format group are presented in Table 1. Significant differences were found between gender format groups in chronological age, anthropometric measures, and aerobic performance, but only aerobic performance showed a significant correlation (r-range: -0.414 to -0.248; p≤0.001) with measures of the intensity of the different game formats: %HRmean and percentage of total time above 80% of maximal HR (%time>80%HRmax). Thus, only aerobic performance measured by distance covered in the Yo-Yo intermittent endurance level 1 test (YYIE1) [21] was used as a covariate in the statistical analysis.

The power calculation for this study was determined post hoc at 5% significance [22]. For all analyzed variables, power for main effects and interaction was above 80%, with the exception of the main effect of gender format on %time>80%HRmax and rating of perceived exertion (RPE) (60 and 45%, respectively) and the main effect of game format on fun (24%).

### 2.2. Experimental Design

In this study, an independent group design was used. The pupils were randomly assigned to same- and mixed-gender game formats. Thus, four gender format groups were studied: girls playing with other girls (G), boys playing with other boys (B), girls playing mixed with boys (GM), and boys playing mixed with girls (BM).

Each of the four gender format groups played one of each of the three selected game formats (2v2, 4v4, and 12v12) on separate days in a random order with at least 48 h in between. The mixed-gender group comprised even representativeness of girls and boys. Since each PE class normally comprises 24-26 pupils, the maximum reasonable number of pupils involved in a single game per team would be 12 to maintain the even representation of genders. Each game lasted 2x15 min, with a 2-min half-time break. The games were played on an artificial grass pitch. The pitch sizes were defined according to typical school settings and aimed to maintain roughly the same area per player and the same length-to-width ratio. Thus, the 2v2, 4v4, and 12v12 games were played on 16x12-m, 23x17-m, and 40x30-m pitches, corresponding to ~50 m^2^ per player and a length-to-width ratio of ~1:0.75. In all game formats, the cone goals were 2 m wide and 0.5 m high and no goalkeepers were allowed. The offside rule was not applied.

### 2.3. Experimental Procedures

The pupils were tested during their weekly PE classes. They were advised to eat a normal diet, including carbohydrates, the day before testing, to eat lunch at least 2 h before testing and to not perform vigorous physical activity on the day before testing. The pupils were also instructed to continue with their usual activity/training schedule during the testing period. All tests were performed after a standardized warm-up wearing the same footwear and under neutral environmental conditions.

HR was recorded at 1-s intervals using HR monitors (Firstbeat Technologies Ltd., Version 4.5.0.2, Jyväskylä, Finland) during the games. Aerobic performance and individual maximal HR (HRmax) were determined beforehand in the YYIE1 using HR monitors. The participants were acquainted with the tests and the use of HR monitors in advance. Internal load during the games was analyzed using HRmean and HRpeak as a percentage of individual HRmax (%HRmean and %HRpeak, respectively), %time>80%HRmax, and RPE.

RPE and perceived level of fun were assessed in a 10 cm visual analogue scale [23] 30 min after the end of all analyzed games in order to ensure that the rating reflected the whole game and not only the final period [24].

Body mass and body fat percentage were measured using a Tanita BC532 InnerScan body composition monitor (Tanita, Amsterdam, Netherlands). Body mass index (BMI) was calculated as the ratio of body mass (kg) to body height squared (m^2^). No maturation status assessment was performed. After being informed about the aims and experimental risks and benefits of the study, the pupils gave their verbal assent and their parents gave their written consent for voluntary participation. The study was conducted in accordance with the Declaration of Helsinki and ethical approval was provided by the local Institutional Review Board.

### 2.4. Statistical Analyses

The results are presented as means±standard deviation (SD) and range, unless otherwise stated. The differences between game and gender formats were examined using a two-way analysis of covariance (ANCOVA) for repeated measures with Bonferroni post hoc multiple-comparison tests, adjusting for aerobic performance. The Pearson correlation coefficient was used to assess the association between the descriptive characteristics of the participants and the intensity of the game formats. Practical significance was assessed by calculating Cohen's* d* effect size and interpreted as suggested by Batterham and Hopkins [25] (*d*≤0.2 trivial,* d*>0.2–0.6 small,* d*>0.6–1.2 moderate,* d*>1.2–2.0 large, and* d*>2.0–4.0 very large). The Student's unpaired* t*-test was used to assess baseline differences in chronological age and anthropometric measures between the four gender game formats. The data were tested for normality using the Shapiro-Wilk test. Statistical Package for the Social Sciences (SPSS Inc., version 23.0) was used for all analyses. The significance level was set at 0.05.

## 3. Results

### 3.1. Main Effects

A main effect of gender format was shown in %HRmean and perceived level of fun (Table 2). The post hoc tests showed lower (*p≤*0.03) %HRmean in GM than in G, B, and BM (71±10 versus 77±7, 77±4, and 74±7%) and less (*p*<0.01) perceived fun in BM than in B, G, and GM (4.4±2.3 versus 6.3±2.3, 6.5±1.9, and 6.6±1.9, respectively).

A main effect of game format was shown in %HRmean, %time>80%HRmax, and RPE (Table 2). Post hoc tests showed no differences between 2v2 and 4v4, but higher (*p*<0.001) values than in 12v12 were observed in %HRmean (78±9 and 76±9 versus 70±10%, respectively), %time>80%HRmax (43±33 and 39±32 versus 23±27%, respectively), and RPE (5.5±2.5 and 5.5±2.7 versus 3.8±2.9, respectively).

### 3.2. Interactions (Gender x Game Formats)

A significant interaction between gender and game formats was found for HRmean, %HRmean, HRpeak, %HRpeak, %time>80%HRmax, RPE, and fun (Table 2).

#### 3.2.1. Comparison of Gender Formats

%HRmean did not differ between gender formats in 2v2 but in 4v4 was lower (*p*=0.02) in GM than in BM (73±12 versus 77±9%;* d*=-0.40, small; Table 2). In 12v12, %HRmean was lower (*p≤*0.01) in GM than in G, B, and BM (63±11 versus 80±8, 73±9 and 69±8%;* d*=-0.63--1.79, moderate-large).

**Figure 1:**
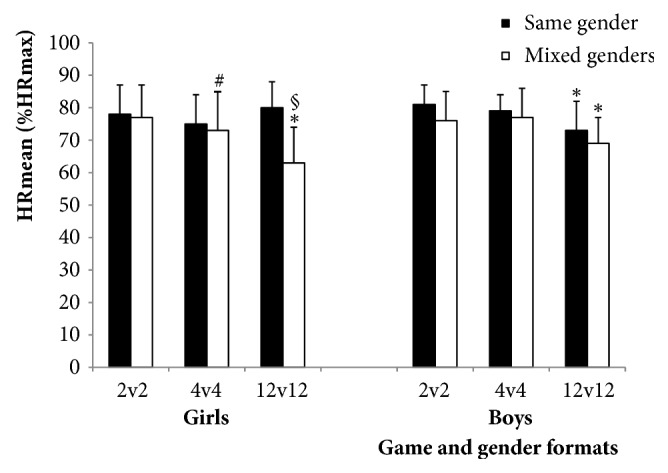
Average heart rate (HRmean) expressed as a percentage of individual maximal HR (%HRmax) values for girls and boys in each gender format (same- and mixed-gender) in the three game formats (2v2, 4v4, and 12v12). Data are presented as means±SD. *∗p*≤0.01 significantly different from 4v4 and 2v2; #*p*=0.02 significantly different from boys in mixed-gender games; §*p*≤0.01 significantly different from girls and boys in same-gender games and boys in mixed-gender games.

**Figure 2:**
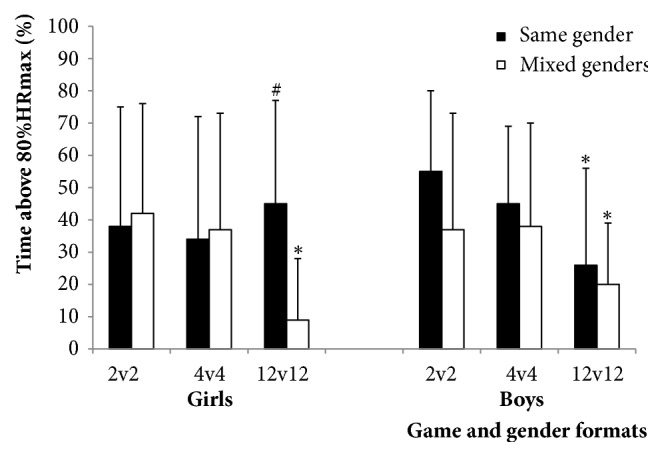
Percentage of total game time spent with heart rate above 80% of individual maximal HR (%HRmax) values for girls and boys in each gender format (same- and mixed-gender) in the three game formats (2v2, 4v4, and 12v12). Data are presented as means±SD. *∗p*<0.01 significantly different from 4v4 and 2v2; #*p*<0.01 significantly different from girls in mixed-gender games.

%time>80%HRmax was higher (*p*<0.01) in G than in GM in 12v12 (45±32 versus 9±19%;* d*=1.41, large), with no significant differences between gender formats in 2v2 and 4v4 (Table 2, Figures 1 and 2).

RPE was lower (*p*<0.01) in G than in GM in 2v2 (3.5±2.5 versus 6.8±2.0;* d=-*1.47, large), whereas no differences between gender formats were observed in 4v4 and 12v12.

Perceived fun was higher (*p*=0.01) in GM than in BM in 4v4 (7.3±2.9 versus 4.9±2.8;* d*=0.84, moderate). In 12v12, BM reported lower (*p*<0.01) perceived fun than B, G, and GM (3.2±2.1 versus 7.0±3.4, 7.3±3.6, and 5.5±3.2;* d*=-0.73–1.22, moderate-large). No differences in perceived fun were found between gender formats in 2v2 (Table 2).

#### 3.2.2. Comparison of Game Formats

%HRmean was higher (*p*<0.01) in 2v2 (76–81%) and 4v4 (73–79%) than in 12v12 (63–73%) in GM, BM, and B (*d*=0.82-1.33, moderate-large; Table 2). No differences between game formats were found in G.

Similarly, %time>80%HRmax was higher (*p*<0.01) in 2v2 (37–55%) and 4v4 (37–45%) than in 12v12 (9–26%) in GM, BM, and B (*d*=0.50-1.25, small-large; Table 2) and no differences between formats were observed in G.

RPE was higher (*p≤*0.05) in 2v2 (5.3–6.8) and 4v4 (4.8–5.7) than in 12v12 (3.4–4.0) in GM and BM (*d*=0.50-1.24, small-large). RPE was higher (*p*=0.01) in 4v4 (5.9±2.5) than in 12v12 (4.1±3.0;* d=*0.64, moderate) in B, whereas RPE in G was higher (*p≤*0.01) in 4v4 (6.3±2.9) than in 2v2 (3.5±2.5;* d*=1.04, moderate) and 12v12 (3.4±3.2;* d*=0.95, moderate; Table 2).

Perceived fun was higher (*p*<0.01) in 2v2 (5.7±2.4;* d*=0.91, moderate) and 4v4 (4.9±2.8;* p*=0.03;* d*=0.58, small) than in 12v12 (3.2±3.1) in BM, whereas no differences were found in perceived fun between game formats for the other gender formats.

## 4. Discussion

The present study describes for the first time the effect of playing same- versus mixed-gender formats on physiological loading and perceived experience of small- and large-sided games.

The main findings of the present study were that HR was lower for girls when they played mixed with boys than when playing alone, whereas HR did not differ for boys whether playing mixed or alone. Moreover, boys perceived less fun when playing mixed with girls than when playing alone, whereas playing alone or mixed with boys had no effect on experienced fun for girls. In addition, game format influenced these observations, with the differences being less apparent in 2v2 and 4v4 than in 12v12. Irrespective of gender, higher HRmean, %time>80%HRmax, and RPE were observed in 2v2 and 4v4 than in 12v12.

The gender format had a large effect on %HRmean (*d*=-1.79) and %time>80%HRmax (*d*=-1.41) for the girls, as lower mean HRs were observed for the girls when playing mixed with the boys than when playing alone in large-sided games (12v12). Interestingly, when comparing girls playing mixed with girls playing alone, HRmean was not lower in 2v2 and 4v4 but there was a large effect in 12v12 (63±11 versus 80±8%HRmax, respectively). In fact, HRmean was lower in 12v12 for girls playing mixed with boys than for any other gender format (moderate to large effect). This could indicate that when the number of participating boys increases, girls are less involved in the game. In boys, %HRmean did not differ depending on whether they played alone or mixed with the girls in any game format.

In a study by Bendiksen and colleagues [16], no differences were observed in HRmean between girls and boys. In that study, small-sided games were organized as 3v3 and no particular attention was paid to gender composition, which may have hidden possible gender differences. The present study did not find differences in HRmean between boys and girls when playing together in 2v2, but a small effect was found in 4v4 and 12v12, with higher HRs shown in boys than in girls when playing mixed-gender game format.

No effect of gender was observed on %time spent >80%HRmax. But in accordance with what we observed for mean and peak HR, less time was spent with high HRs (i.e., >80%HRmax) for girls when playing mixed with boys in 12v12 (9% of the total time) than when playing alone (45% of the total time; large effect).

RPE did not differ between genders, but in 2v2 the girls perceived the game to be much harder when playing mixed with boys than when playing alone (large effect). RPE has been shown to correlate with cardiovascular load (HR) in adult male soccer players [26], but in this study no difference was found in mean or peak HR or in %time>80%HRmax between girls playing alone and mixed with boys in 2v2, so the higher perceived exertion cannot be explained by differences in cardiovascular load. Interestingly, a moderate effect on fun score was found between girls playing alone and mixed in 2v2, with a tendency for a slightly higher (*p*>0.05) fun score when playing mixed with boys.

Boys reported a lower fun score when playing mixed with girls than when playing alone in 12v12 (*d*=-1.17; moderate effect), whereas no such difference was seen for girls. In this game format, the effect on fun score was moderate to large between boys playing mixed and any other gender format, showing lower values. No difference between gender formats in respect of fun was found in 2v2, but in 4v4 girls playing mixed reported higher fun scores than boys playing mixed, while in 12v12 boys playing mixed reported less fun than any other gender format (moderate to large effect). Interestingly, fun scores tended to increase with an increasing number of players when boys played alone (thus considering 12v12 more fun than 2v2), whereas when playing with the girls, fun decreased for the boys when the number of players, and hence the number of girls, increased (small to moderate effect).

Taken together, these results suggest that if the purpose is to improve cardiorespiratory fitness or promote enjoyment, boys and girls should play separately, as girls showed higher HRs when playing alone than when playing with the boys and the boys reported higher fun scores when playing alone than when playing mixed. The differences were more pronounced in the large-sided format (12v12) than in the small-sided formats (2v2 and 4v4).

Regarding the effect of the game format (e.g., number of players), higher HRmean values were observed in small-sided (2v2 and 4v4) than in large-sided (12v12) games. When girls played mixed-gender games and when boys played mixed- as well as same-gender games, the aerobic exercise intensity (%HRmean and %time>80%HRmax) was much higher during small-sided games (2v2 and 4v4) than during large-sided games (12v12) (small to large effect). Previous studies involving untrained male adults showed no differences in HR response between 7v7 compared with 4v4 and small-sided games with fewer players, whereas female players showed small differences [18]. The higher HRs in game formats with fewer players are in line with some studies [27–29], whereas others did not find any effects of the number of players [30–32]. HRmean during the games was 63–81%HRmax, which, except for mixed-gender 12v12 (GM: 63, and BM: 69%HRmax), is similar to what has previously been observed (HRmean 71–79%HRmax) in younger schoolchildren (8–13-year-olds) in small-sided soccer games [16, 18, 19] and other team-sport activities [16]. %time>80%HRmax was lower (39–43% versus 51–57%) [18], but HRmean values in 2v2 and 4v4 were still above the levels shown to induce functional and structural cardiac adaptations [19] and within the range (74–78%HRmax) shown to improve cardiorespiratory fitness [16] in children using 3v3 small-sided soccer games in a short-term school-based intervention. Cardiorespiratory fitness has been identified as a strong independent predictor of risk of cardiovascular diseases and mortality [33, 34]. Additionally, there is a growing body of evidence of the positive health and fitness effects of soccer-based interventions in children [14, 16, 19, 35, 36] analogous with those observed in adults [5, 18], and this study provides further information on which game and gender formats can elicit the high HRs necessary for such improvements. In fact, spending 20–29% (10–14 min) of total playing time with HR above 90%HRmax is considered sufficient to cause marked improvements in cardiorespiratory fitness, systolic blood pressure, and glucose tolerance [37] and to thereby increase overall health profile in adults in soccer-based training interventions (2–3 times per week). In children, a 22% improvement in cardiorespiratory fitness was shown after 6 weeks of 3v3 soccer and unihockey comprising ~30 min sessions two times per week with only 9–12% of total time with HR above 90%HRmax [16]. In the present study, %time>90% was 11–13% of total game time for 2v2 and 4v4, but only 5% for 12v12 (data not shown), and higher %time>80%HRmax was observed in 2v2 and 4v4 (39–43% of total time) than in 12v12 (23% of total time; small to large effect). Furthermore, mean HRs in 12v12 (70%HRmax) were lower than in 11v11 matches (~80%HRmax) reported for recreational 12-year-old soccer players [38] and 11–12-year-old elite and recreational soccer players [39]. Nonetheless, for 2v2 and 4v4 game formats, HRmean values (78 and 76%, respectively) and %time>80%HRmax (43 and 39%, respectively) were higher than values shown to improve cardiac structure and function (71% HRmean; 24% time >80%HRmax) in children using 3x40-min 3v3 soccer training sessions per week including warm-up and technical drills over 8–10 weeks and similar to 12v12 (70% HRmean; 23% time >80%HRmax) [19].

Despite the relatively high HRs, the fun scores were moderate to high (4.9–7.3) in the different game and gender formats, with the exception of 12v12 for boys playing mixed with girls (3.2, 0–10 scale). This highlights the potential of soccer for keeping pupils motivated while performing a highly demanding activity, which is of paramount importance for health and fitness adaptations. Studies have shown greater involvement during games with fewer players [39]. If the players are more involved in the game, there will be more situations for decision-making, which may lead to increased skill acquisition and thereby enjoyment [40]. This could motivate the children to engage in soccer practice in an organized setting outside school. This is important because soccer participation has been associated with decreased time spent in sedentary activity and increased time spent in moderate-to-vigorous physical activity, and children playing soccer at any frequency have demonstrated 3 to 15 times increased odds of achieving at least 60 min of moderate-to-vigorous physical activity per day compared to children not participating in organized sports [41]. Also, it is noteworthy that for girls, a high intensity can be maintained or increased during a 30-min period with 2-min half-time break, while for boys match intensity decreases in the second half (data not shown).

This study has a number of strengths and limitations that should be acknowledged. It is a strength of the study that it describes the physiological demands and perceived experience of adolescent pupils playing different gender formats. Secondly, unlike most previous studies [17, 39], standardized conditions were used during the games (e.g., area per player, length-to-width pitch ratio, teacher encouragement, rules of the game, goal size, and absence of goalkeepers), allowing a better understanding of the isolated role of the two factors under analysis, number of players, and gender format. With regard to limitations, it should be mentioned that no locomotor activity analyses were performed during the various game formats. Previous studies have shown similar HRs in different small-sided games, though the number of high-intensity actions and bouts was different [18]. Nevertheless, Randers and colleagues [42] found no significant effect of the number of players on distance covered in sprinting and high-intensity running. Additionally, the number of technical actions was markedly higher during the game formats with fewer players, with the greatest effect of game format for the players with low technical involvement, which is of importance in school settings [39]. This study partly supports this, as BM reported more fun in 2v2 (5.7±2.4;* p*<0.01;* d*=0.91) and 4v4 (4.9±2.8;* p*=0.03;* d*=0.58) than in 12v12 (3.2±3.1), showing a small-to-moderate effect. Further studies are warranted to describe the fitness and health effects of using same- and mixed-gender formats with small- and large-sided games in short- and long-term randomized controlled school-based interventions. Additionally, although chronological age and anthropometric measures were not associated with the measures of the intensity of the different game formats, future studies with a larger sample size allowing for differential chronological age and anthropometric variables analysis across gender and game formats should be carried out.

In summary, aerobic exercise intensity was much higher with small-sided than with large-sided games when girls played mixed-gender games and when boys played mixed- and same-gender games. In general, small-sided soccer can be recommended for adolescent girls and boys when the intention is to improve cardiorespiratory fitness or to promote enjoyment.

## Figures and Tables

**Table 1:** Chronological age, anthropometric characteristics, and aerobic performance of the participants by gender group format (means±SD and range).

Gender group	Age	Body mass	Stature	Fat mass	BMI	YYIE1
	(years)	(kg)	(cm)	(%)	(kg.m^−2^)	(m)
Girls (*n* = 22)	14.2 ± 0.7 (13-15)^c,d^	58.9 ± 9.1 (45.1-80.6)	161.5 ± 6.2 (151.0-173.0)	28.0 ± 5.8 (12.4-39.6)^b,d^	22.4 ± 2.8 (19.1-26.6)	851 ± 559 (320-2200)^d^
Boys (*n* = 38)	14.0 ± 0.8 (13-16)^c,d^	58.7 ± 12.6 (36.9-97.7)^d^	164.3 ± 7.8 (149.0-183.0)^c^	18.6 ± 7.2 (8.7-39.9)^a,c^	21.7 ± 3.9 (15.8-35.9)	1048 ± 711 (160-2680)^d^
Girls mixed (*n* = 28)	12.8 ± 0.7 (12-14)^a,b^	52.0 ± 9.2 (36.2-76.5)	157.5 ± 7.2 (141.0-168.0)^b^	25.5 ± 6.2 (16.7-43.6)^b,d^	20.9 ± 3.5 (16.9-31.8)	945 ± 557 (200-2320)^d^
Boys mixed (*n* = 46)	13.0 ± 0.8 (12-15)^a,b^	51.6 ± 10.3 (34.2-72.9)^b^	159.8 ± 9.4 (144.0-184.0)	15.8 ± 6.6 (7.5-40.4)^a,c^	20.1 ± 2.9 (15.1-29.4)	1583 ± 846 (160-3600)^a,b,c^

BMI = body mass index and YYIE1 = Yo-Yo intermittent endurance level 1 test, significantly different (*p* ≤ 0.02) from girls (a), boys (b), girls mixed (c), and boys mixed (d).

**Table 2:** Game intensity and perceived experience. Values are *F*-test and *p* value for analysis of covariance (ANCOVA) model by game and gender format and controlled for aerobic performance.

													Two-way ANCOVA		
Gender format	Girls (*n* = 22)			Girls mixed (*n* = 38)			Boys (*n* = 28)			Boys mixed (*n* = 46)			Gender format	Game format	Interaction
Game format	2v2	4v4	12v12	2v2	4v4	12v12	2v2	4v4	12v12	2v2	4v4	12v12	F(df; error); p	F(df; error); p	F(df; error); p
HRmean (b.min^−1^)	154 ± 21	150 ± 22	159 ± 18	159 ± 21	152 ± 25	130 ± 23	163 ± 13	159 ± 10	146 ± 19	154 ± 18	157 ± 18	141 ± 16	2.444(3;178); 0.069	13.460(2;178); 0.000	7.095(6;178); 0.000
HRmean (%HRmax)	78 ± 9	75 ± 9	80 ± 8	77 ± 10	73 ± 12	63 ± 11	81 ± 6	79 ± 5	73 ± 9	76 ± 9	77 ± 9	69 ± 8	5.455(3;178); 0.002	13.264(2;178); 0.000	7.064(6;178); 0.000
HRpeak (b.min^−1^)	179 ± 19	177 ± 20	183 ± 16	185 ± 18	179 ± 24	164 ± 27	190 ± 10	188 ± 8	181 ± 15	183 ± 16	184 ± 15	178 ± 16	3.986(3;178); 0.010	6.568(2;178); 0.002	3.984(6;178); 0.001
HRpeak (%HRmax)	90 ± 7	89 ± 8	92 ± 7	89 ± 9	86 ± 11	79 ± 13	94 ± 4	93 ± 4	90 ± 7	90 ± 7	90 ± 7	87 ± 7	8.840(3;178); 0.000	6.450(2;178); 0.002	3.979(6;178); 0.001
%Time>80% HRmax (%)	38 ± 37	34 ± 38	45 ± 32	42 ± 34	37 ± 36	9 ± 19	55 ± 25	45 ± 24	26 ± 30	37 ± 36	38 ± 32	20 ± 19	1.631(3;178); 0.188	14.162(2;178); 0.000	4.152(6;178); 0.001
RPE (AU, 0-10 scale)	3.5 ± 2.5	6.3 ± 2.9	3.4 ± 3.2	6.8 ± 2.0	5.7 ± 2.7	4.0 ± 2.5	5.4 ± 2.5	5.9 ± 2.5	4.1 ± 3.0	5.3 ± 2.4	4.8 ± 2.6	3.4 ± 3.0	1.126(3;170); 0.343	5.543(2;178); 0.005	3.095(6;170); 0.007
Fun (AU, 0-10 scale)	5.4 ± 2.3	6.7 ± 2.1	7.3 ± 3.6	6.9 ± 2.8	7.3 ± 2.9	5.5 ± 3.2	5.4 ± 3.3	6.0 ± 2.8	7.0 ± 3.4	5.7 ± 2.4	4.9 ± 2.8	3.2 ± 3.1	8.279(3;170); 0.000	1.356(2;178); 0.260	3.675(6;170); 0.002

%time>80%HR_max_ = percentage of time above 80%HRmax and AU = arbitrary units.

## Data Availability

The data used to support the findings of this study are available from the corresponding author upon reasonable request.
